# Noninvasive Screening Tool for Hyperkalemia Using a Single-Lead Electrocardiogram and Deep Learning: Development and Usability Study

**DOI:** 10.2196/34724

**Published:** 2022-06-03

**Authors:** Erdenebayar Urtnasan, Jung Hun Lee, Byungjin Moon, Hee Young Lee, Kyuhee Lee, Hyun Youk

**Affiliations:** 1 Artificial Intelligence Big Data Medical Center Wonju College of Medicine Yonsei University Wonju Republic of Korea; 2 Bigdata Platform Business Group Yonsei Wonju Health System Wonju Republic of Korea; 3 Department of Emergency Medicine Wonju College of Medicine Yonsei University Wonju Republic of Korea; 4 Center of Regional Trauma Wonju Republic of Korea

**Keywords:** hyperkalemia, ECG, electrocardiogram, deep learning, noninvasive screening, emergency medicine, single-lead ECG

## Abstract

**Background:**

Hyperkalemia monitoring is very important in patients with chronic kidney disease (CKD) in emergency medicine. Currently, blood testing is regarded as the standard way to diagnose hyperkalemia (ie, using serum potassium levels). Therefore, an alternative and noninvasive method is required for real-time monitoring of hyperkalemia in the emergency medicine department.

**Objective:**

This study aimed to propose a novel method for noninvasive screening of hyperkalemia using a single-lead electrocardiogram (ECG) based on a deep learning model.

**Methods:**

For this study, 2958 patients with hyperkalemia events from July 2009 to June 2019 were enrolled at 1 regional emergency center, of which 1790 were diagnosed with chronic renal failure before hyperkalemic events. Patients who did not have biochemical electrolyte tests corresponding to the original 12-lead ECG signal were excluded. We used data from 855 patients (555 patients with CKD, and 300 patients without CKD). The 12-lead ECG signal was collected at the time of the hyperkalemic event, prior to the event, and after the event for each patient. All 12-lead ECG signals were matched with an electrolyte test within 2 hours of each ECG to form a data set. We then analyzed the ECG signals with a duration of 2 seconds and a segment composed of 1400 samples. The data set was randomly divided into the training set, validation set, and test set according to the ratio of 6:2:2 percent. The proposed noninvasive screening tool used a deep learning model that can express the complex and cyclic rhythm of cardiac activity. The deep learning model consists of convolutional and pooling layers for noninvasive screening of the serum potassium level from an ECG signal. To extract an optimal single-lead ECG, we evaluated the performances of the proposed deep learning model for each lead including lead I, II, and V1-V6.

**Results:**

The proposed noninvasive screening tool using a single-lead ECG shows high performances with F1 scores of 100%, 96%, and 95% for the training set, validation set, and test set, respectively. The lead II signal was shown to have the highest performance among the ECG leads.

**Conclusions:**

We developed a novel method for noninvasive screening of hyperkalemia using a single-lead ECG signal, and it can be used as a helpful tool in emergency medicine.

## Introduction

Hyperkalemia is a potential life-threatening condition for the general population, and so it can be a clinical and economic burden [1]. Normal levels of potassium are between 3.5 and 5.0 mmol/L with levels above 5.5 mmol/L defined as hyperkalemia. Patients with chronic kidney disease (CKD) are predisposed to hyperkalemia [2], and it is a major risk factor for cardiac arrhythmias and death [3]. According to some clinical studies, serum potassium monitoring can reduce the risk of hyperkalemia in patients with CKD by more than 71% [4]. Therefore, it is very important to frequently check the serum potassium level in patients with CKD.

Potassium is a very important electrolyte for the regulation of the cell membrane potential and nerve conduction, so abnormal levels of potassium are known to be associated with changes in electrocardiogram (ECG) readings. Hyperkalemia is associated with tall, narrow, and symmetrical T waves in an ECG, whereas hypokalemia is associated with flat T waves [5-9]. Monitoring hyperkalemia is very important in patients with CKD, but so far blood testing is the only way to test serum potassium levels. Closer and more reliable monitoring requires the development and verification of noninvasive and continuous monitoring methods.

Electrocardiography is used to detect heart abnormalities in patients with various diseases. The main ECG changes associated with hypokalemia include a decreased T wave amplitude, ST-segment depression, T wave inversion, a prolonged PR interval, and an increased corrected QT interval [10]. The typical ECG findings for hyperkalemia progress from tall, peaked T waves and a shortened QT interval to a lengthened PR interval and a loss of the P wave followed by a widening QRS complex and ultimately a sine wave morphology [11,12]. These morphologic differences of the ECG have been used to detect and diagnose hyperkalemia events urgently in emergency rooms [13]. In addition, there are some studies that have proposed several methods to detect hyperkalemia events using ECG signals. Among them, some researchers have developed ECG quantification algorithms to predict serum potassium concentration based on T wave morphology, mainly using the slope and width of T waves. The algorithms were mostly derived from continuous patient monitoring, such as during hemodialysis, with homogeneous ECG morphologies from a limited set of patients [14,15]. Recently, applying the processing of T wave morphologies manually has been used to improve the diagnosis of hyperkalemia [16]. Nevertheless, using T wave changes alone to detect dyskalemias is less sensitive and specific than a comprehensive ECG interpretation [17]. However, ECG morphology–based methods have shown insufficient performance and require some time to extract the morphologic features required to detect hyperkalemia. Therefore, a more robust and faster method for hyperkalemia detection is needed in the clinical practice of emergency medicine.

With the revolution of artificial intelligence (AI), many deep learning models have been developed that show human-level performance in several clinical fields such as cardiology [18], radiology [19], ophthalmology [20], and pathology [21]. For instance, convolutional neural network (CNN) models have achieved very high performances for abnormal cardiac rhythms such as arrhythmia [22], tachycardia [23], and supraventricular dysfunction [24], among other events [25]. Such diagnostic and prognostic deep learning models could be developed to assist emergency medicine clinicians in recognizing ECG changes associated with diverse diseases. AI algorithms have emerged in clinical decision support systems as “software as a medical device” in a real clinical environment [26]. There are some similar studies conducted by several researchers. Among them, Galloway et al [11] proposed a CNN-based model to screen for hyperkalemia using a multilead ECG signal. They demonstrated a deep CNN model with complex architecture and evaluated its performance using big ECG data sets in a multicenter cohort study. Another study involved the prediction of serum potassium concentration based on an 82-layer CNN model using a 12-lead ECG signal [12]. They achieved robust performance with more than 95.8% hyperkalemia detection. However, all these recent deep learning models are hard to apply to real-time analysis in clinical practices.

Therefore, this study proposed a novel method for noninvasive screening of hyperkalemia based on a deep learning model using an ECG. For this purpose, we constructed a deep learning structure using a CNN model, and clinical data were used for the training and testing phases. In addition, we conducted several experiments using the different data sets, applying the changes before and after hyperkalemia events. Finally, a simple and accurate deep learning model was designed to implement a noninvasive screening method for hyperkalemia that can be applied to real-time clinical practices.

## Methods

### Overview

**Figure 1:**
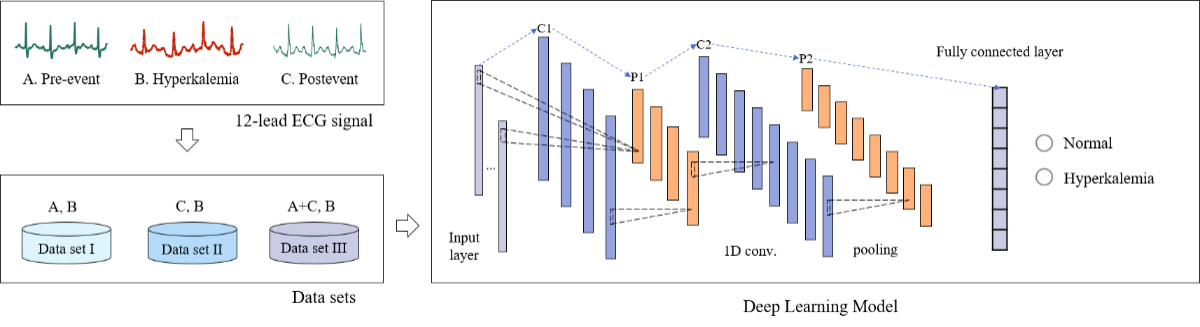
Diagram of the proposed method for noninvasive screening of hyperkalemia. ECG: electrocardiogram.

In this study, we proposed a novel method for noninvasive screening of hyperkalemia based on a deep learning model, using ECG. The proposed method consists of the following 3 main parts: 12-lead ECG extraction from the participants, constructing the ECG data sets, and a deep learning model (Figure 1). Each part of the study method is explained in more detail in the following subsections.

### Ethics Approval

This study was approved by the Institutional Review Board (IRB) of the Wonju Severance Christian Hospital (CR320162). Enrolled patients’ informed consent was exempted by the IRB due to the retrospective nature of the study that used fully anonymized ECG and health data.

### Participants

A total of 2958 patients who have experienced at least 1 hyperkalemia event were enrolled at a single regional emergency center from July 2009 to June 2019. Among them, 1790 patients were diagnosed with chronic renal failure (CRF), and the other 1168 patients did not have CRF. The patients who did not have biochemical electrolyte tests corresponding to the original 12-lead ECG signal were excluded. We then used the data of 855 patients (555 patients with CRF, 300 patients without CRF) (Table 1).

**Table 1:** Characteristics of the study participants.

Characteristics		Participants (N=2958)		
		Non-CRF^a^ (n=1168)	CRF (n=1790)	Total
**Gender, n (%)^b^**				
	Female	496 (39.9)	747 (60.1)	1243 (42)
	Male	672 (39.2)	1043 (60.8)	1715 (58)
	Total	1168 (39.5)	1790 (60.5)	2958 (100)
Age (years), mean (SD)		70.3 (19.0)	72.6 (13.2)	71.7 (15.8)
Height (cm), mean (SD)		155.5 (26.2)	159.4 (14.4)	158.0 (19.7)
Weight (kg), mean (SD)		58.8 (15.5)	62.2 (12.2)	60.9 (13.6)
Myocardial infarction, n (%)^b^		35 (23)	117 (77)	152 (5.1)
Heart failure, n (%)^b^		116 (30)	271 (70)	387 (13.1)
Angina, n (%)^b^		93 (28.4)	235 (71.6)	328 (11.1)
Diabetes, n (%)^b^		251 (21.6)	912 (78.4)	1163 (39.3)
Hypertension, n (%)^b^		323 (23.8)	1037 (76.3)	1360 (46)

^a^CRF: chronic renal failure.

^b^The denominator used to calculate percentages is the sum of the non-CRF and CRF participants in that category (ie, row).

### Data Sets

The 12-lead ECG recordings were collected at the 3 sections of hyperkalemic events: pre-event, event, and postevent. The pre- and postevents were used as normal or control events, and the hyperkalemic event was used as the target or abnormal event. From these 3 sections, the data sets were designed, including data set I (pre-event vs event), data set II (postevent vs event), and data set III (pre-event and postevent vs event). The differences between before- and aftereffects of a hyperkalemia event are presented in Table 2. All 12-lead ECG recordings were matched with an electrolyte test within 2 hours in each section to form a data set. The waveform of the 12-lead ECG signal was then extracted and saved with a sampling frequency of 700 Hz. Finally, an ECG signal segment with a duration of 2 seconds was composed of 1400 samples. For evaluation of the developed AI algorithm, the ECG data set was randomly divided by the ratio of 6:2:2 percent into the training set, validation set, and test set for each data set.

**Table 2:** Data sets for this study.

Data sets	Data set I, n	Data set II, n	Data set III, n
Training set	1186	879	1426
Validation set	296	220	357
Test set	370	275	446
Total	1852	1374	2229

### Deep Learning Model

Deep learning is a method for representation learning that can learn the complex pattern and structure of the input data by high-level data abstraction. It can learn the morphology of the input ECG signal according to the potassium concentrations. A deep learning model was designed based on a 5-layer CNN by using a 1-dimensional convolutional operation, max pooling, and a fully connected layer. The detailed structure of the proposed deep learning model for noninvasive screening of hyperkalemia using ECG signal is shown in Table 3.

**Table 3:** Architecture of the proposed deep learning model for hyperkalemia screening.

Number and layers			Activation	Filter size		Output shape		Parameter
**1**								
	batchnorm_1	=		=	1400×1		4	
**2**								
	conv1D_1 maxpool_1	ReLu		100@50×1 2×1	1351×100 675×100		5100	
**3**								
	conv1D_2 maxpool_2 dropout_2	ReLu		80@50×1 2×1 p=0.25^a^	626×80 313×80 313×80		400,080	
**4**								
	conv1D_3 maxpool_3 dropout_3	ReLu		60@30×1 2×1 p=0.25	284×60 142×60 142×60		144,060	
**5**								
	conv1D_4 maxpool_4 dropout_4	ReLu		40@20×1 2×1 p=0.25	123×40 61×40 61×40		48,040	
**6**								
	conv1D_5 maxpool_5 dropout_5	ReLu		20@10×1 2×1 p=0.25	52×20 26×20 26×20		8020	
**7**								
	flatten_1 dense_1	Softmax		2	520×2		1042	
Total	5 conv layers			124 filters			606,027	

^a^*p*: One of the setting parameters of the dropout technique.

### Statistical Analysis

The F1 score was used to evaluate the proposed noninvasive screening method for hyperkalemia; it evaluates the correct classification of each class according to class equality. F1 scores calculated by precision and recall are represented as follows:



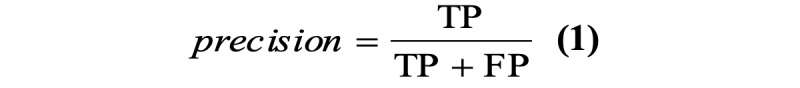





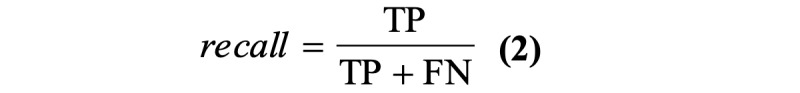



The numbers of true positives (TP), false positives (FP), and false negatives (FN) are input into the equations. The F1 score is computed based on the sample proportion of precision and recall as follows:



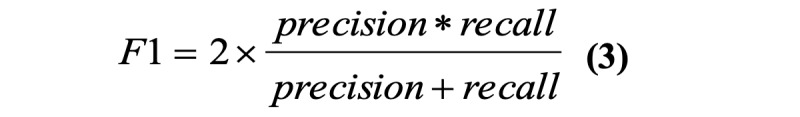



## Results

The results of the proposed novel method for noninvasive screening of hyperkalemia based on deep learning using a 12-lead ECG are shown in Table 4-Table 6 for the test set of each data set. The results of the proposed method showed that lead II achieved the highest performance for hyperkalemia events among other leads for data set I (Table 4).

**Table 4:** The performance of the proposed method for the test set of data set I.

Index and events		Leads							
		I	II	V1	V2	V3	V4	V5	V6
**Precision**									
	Normal	0.52	0.96	0.47	0.61	0.56	0.66	0.51	0.54
	Hyperkalemia	0.61	0.94	0.63	0.70	0.63	0.71	0.64	0.63
**Recall**									
	Normal	0.48	0.93	0.56	0.66	0.51	0.66	0.50	0.60
	Hyperkalemia	0.64	0.97	0.54	0.65	0.68	0.71	0.65	0.58
**F1 score**									
	Normal	0.50	0.94	0.51	0.64	0.53	0.66	0.50	0.57
	Hyperkalemia	0.62	0.95	0.58	0.68	0.66	0.71	0.65	0.60

**Table 5:** The performance of the proposed method for the test set of data set II.

Index and events			Leads														
			I		II		V1		V2		V3		V4		V5		V6
**Precision**																	
	Normal	0.31		0.88		0.28		0.36		0.28		0.52		0.36		0.51	
	Hyperkalemia	0.75		0.93		0.74		0.73		0.80		0.79		0.76		0.72	
**Recall**																	
	Normal	0.22		0.85		0.17		0.27		0.23		0.50		0.28		0.30	
	Hyperkalemia	0.82		0.95		0.84		0.81		0.84		0.81		0.82		0.87	
**F1 score**																	
	Normal	0.26		0.87		0.21		0.31		0.25		0.51		0.31		0.38	
	Hyperkalemia	0.78		0.94		0.79		0.77		0.82		0.80		0.79		0.79	

**Table 6:** The performance of the proposed method for the test set of data set III.

Index and events		Leads							
		I	II	V1	V2	V3	V4	V5	V6
**Precision**									
	Normal	0.56	0.95	0.53	0.68	0.65	0.69	0.57	0.61
	Hyperkalemia	0.47	0.94	0.74	0.59	0.57	0.60	0.60	0.51
**Recall**									
	Normal	0.61	0.96	1.00	0.70	0.62	0.63	0.68	0.59
	Hyperkalemia	0.42	0.93	0.00	0.57	0.60	0.66	0.48	0.53
**F1 score**									
	Normal	0.58	0.96	0.70	0.69	0.64	0.66	0.62	0.60
	Hyperkalemia	0.44	0.94	0.00	0.58	0.59	0.63	0.53	0.52

We also noticed that there are big performance differences between lead II and other leads not only in data set I, but also in data sets II and III. The results showed that V2 achieved the best performance among the V1-V6 leads throughout the 3 different data sets.

We obtained good performances of the proposed deep learning model for noninvasive screening of hyperkalemia using lead II signal, with F1 scores of 95%, 94%, and 94% for data set I, data set II, and data set III for the test set.

**Figure 2:**
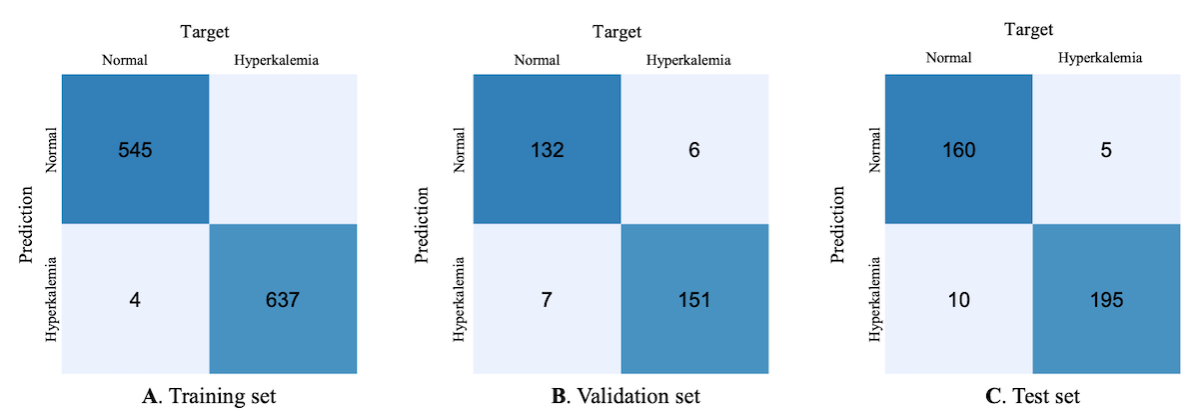
Confusion matrix of this study. Confusion matrix of (A) the training set, (B) the validation set, and (C) the test set for the lead II electrocardiogram channel of data set I.

For data set I, we presented the confusion matrix of the training set, validation set, and test set for lead II of the ECG. The results showed good performance, with F1scores of 100%, 96%, and 95% for the training set, validation set, and test set, respectively. The confusion matrix showed that the proposed deep learning model gained a high and stable rate for the true positives and false negatives (Figure 2).

## Discussion

In this study, we demonstrated a novel method for noninvasive screening of hyperkalemia based on deep learning using ECG. We designed an optimal and simple architecture of deep learning that can be easily implemented for real clinical applications, especially in emergency medicine. The proposed model achieved good performances based on feature extraction of the characteristics of cardiac activity according to the levels of electrolytes using ECG.

We have tried to see the morphological or rhythmical differences between hyperkalemia pre- and postevents in ECG waveform. To do this, we set up 3 different data sets that selected from the pre-event, postevent, and target event sections: data set I (pre-event vs event), data set II (postevent vs event), and data set III (pre- and postevent vs event). All data sets were applied to the designed deep learning model for hyperkalemia screening in training, validation, and test phases. We trained and tested the 3 different deep learning models using each data set. We also conducted experiments to find the optimal signal of the 12-lead ECG for each data set one by one. We determined that an optimal lead for hyperkalemia screening was lead II for our 12-lead ECG data sets. In general, lead II contains the most information on cardiac activity among the leads, which may have resulted in it having the highest performance for the hyperkalemia screening. In addition, our results support conventional studies on hyperkalemia screening using ECG signals.

There are some similar studies that proposed a screening or detection tool for hyperkalemia using ECG signals. The earliest one was proposed by Wrenn et al [10] in 1991. This study compared hyperkalemia detection by 2 independent and experienced physicians using ECG signals. The results showed a sensitivity of 62.0% for the first reader and 55.0% for the second reader and the κ value was 0.73. This showed how difficult it was to detect hyperkalemia from the ECG signal without any biochemical electrolyte tests. Another study on the correlation between hyperkalemia and ECG morphologies was conducted by Levis [27] in 2013. They published a clinical case study on ECG signals observed as a hyperkalemia event occurs. The authors demonstrated 2 different cases of older adults with acute renal failure and hyperkalemia. They confirmed the ECG morphology changes with peaked T waves, shortened QT interval, and lengthening PR intervals corresponding to the hyperkalemia or changes of serum potassium levels.

Recently, deep learning models have been applied to studies on detection and screening of hyperkalemia from the 12-lead ECG recordings made in emergency departments. Galloway et al [11] developed and validated a deep learning model to screen for hyperkalemia using ECG. The authors designed an 11-layer deep CNN model, and it was trained and validated with 449,380 patients with CKD. They used 2 data sets for 2 leads (I and II) and 4 leads (I, II, V3, and V5); each patient had a serum potassium count drawn within 4 hours after their ECG was recorded. In this multicenter cohort study, a deep learning model was developed with complex architecture, using big ECG data sets [11]. However, they achieved a good performance, with an area under the curve (AUC) of 88.83% and a sensitivity of 91.3% for the 2 leads’ data sets. In contrast, we proposed a deep learning model with a light weight and high performance using a single-lead ECG signal.

Lin et al [12] developed a 12-channel sequence-to-sequence model with an 82-layer CNN structure to predict serum potassium concentration by using a 12-lead ECG signal. They modified DenseNet architecture to read the 12-lead ECG waveforms and detect hypokalemia and hyperkalemia events, and named it ECG12Net. ECG12Net achieved robust performances, with an AUC of 95.8% and 97.6% for hyperkalemia and severe hyperkalemia, respectively. However, ECG12Net requires very high computational power to read the 12-lead ECG signal since it is composed of an 82-layer CNN model. Our method is comparable in performance with this model, and it is well optimized and trained for a 1-channel ECG signal to detect hyperkalemia events. In addition, it is easy to make it into a tool that can be used in the final clinical field because the deep learning engine is relatively lighter.

The proposed new method for noninvasive screening of hyperkalemia based on deep learning using ECG signals surpasses similar previous studies, and the developed model can be applied directly to clinical situations. This noninvasive method does not require any blood test or invasive chemistry diagnosis. In addition, the physicians or clinicians can check the results quickly, within 3 minutes, which is faster than previous invasive diagnostic methods. This is because the deep learning model has a simple structure and is well optimized and trained by the pre- and postevent’s ECG signals to screen for hyperkalemia events. In addition, the proposed deep learning model can proceed with feature extraction and classification at once from the input ECG signal for noninvasive screening of hyperkalemia because we do not use any handcrafted preprocessing that extracts input ECG components such as the RR interval or P wave. Finally, we achieved a higher performance of the proposed noninvasive method based on a deep learning model; it can consider the complex and cyclic characteristics of ECG affected by levels of electrolytes.

This study has some limitations, such as the small study population, and there are many comorbidities including heart failure, diabetes, and hypertension in the study groups. In addition, all participants of this study are enrolled at a single regional emergency center, so further study should cover large and diverse populations from multiple centers.

We demonstrated a novel method for noninvasive screening of hyperkalemia based on deep learning using ECG. We obtained high performances with an F1 score of 95% from the ECG signal. In addition, we developed a simple and accurate deep learning model for the noninvasive screening of hyperkalemia that can be used in real-time clinical settings. Therefore, the proposed deep learning model may be appropriate for the noninvasive screening of hyperkalemia using a single-lead ECG signal without any feature extraction (eg, T wave and QT interval). Furthermore, a validation study should be conducted for the proposed deep learning model that uses larger and more diverse data sets based on a single-lead ECG signal.

## Abbreviations

AI: artificial intelligence
AUC: area under the curve
CKD: chronic kidney disease
CNN: convolutional neural network
ECG: electrocardiogram
IRB: Institutional Review Board
